# DOK3 Negatively Regulates LPS Responses and Endotoxin Tolerance

**DOI:** 10.1371/journal.pone.0039967

**Published:** 2012-06-27

**Authors:** Qisheng Peng, Jason L. O’Loughlin, Mary Beth Humphrey

**Affiliations:** 1 Department of Medicine, University of Oklahoma Health Sciences Center, Oklahoma City, Oklahoma, United States of America; 2 Key Laboratory for Zoonosis Research, Ministry of Education, Jilin University, Changchun, China; 3 Department of Veterans Affairs, Oklahoma City, Oklahoma, United States of America; Charité-University Medicine Berlin, Germany

## Abstract

Innate immune activation via Toll-like receptors (TLRs), although critical for host defense against infection, must be regulated to prevent sustained cell activation that can lead to cell death. Cells repeatedly stimulated with lipopolysaccharide (LPS) develop endotoxin tolerance making the cells hypo-responsive to additional TLR stimulation. We show here that DOK3 is a negative regulator of TLR signaling by limiting LPS-induced ERK activation and cytokine responses in macrophages. LPS induces ubiquitin-mediated degradation of DOK3 leading to SOS1 degradation and inhibition of ERK activation. DOK3 mice are hypersensitive to sublethal doses of LPS and have altered cytokine responses in vivo. During endotoxin tolerance, DOK3 expression remains stable, and it negatively regulates the expression of SHIP1, IRAK-M, SOCS1, and SOS1. As such, DOK3-deficient macrophages are more sensitive to LPS-induced tolerance becoming tolerant at lower levels of LPS than wild type cells. Taken together, the absence of DOK3 increases LPS signaling, contributing to LPS-induced tolerance. Thus, DOK3 plays a role in TLR signaling during both naïve and endotoxin-induced tolerant conditions.

## Introduction

Toll-like receptors (TLRs) are pattern recognition receptors used by cells of the innate immune system to detect pathogen-associated molecular patterns (PAMPs), including the bacterial cell wall component lipopolysaccharide (LPS), which combines with the small molecule MD2 to activate TLR4 [1]. LPS, in turn, initiates downstream intracellular signaling events, including the activation of NF-κB and of the mitogen activated protein kinases (MAPKs) ERK, JNK, and p38 [1], [2], [3], [4]. Activation of these signaling components leads to the production of pro-inflammatory cytokines, including tumor necrosis factor α (TNFα), interleukin-1β (IL-1β), and interleukin-6 (IL-6).

Although TLRs are essential for initiating activation of innate defenses and for enhancing adaptive responses to pathogens, inappropriate regulation of cytokine production contributes to chronic inflammation and to systemic autoimmune diseases [5]. In septic patients and experimental animal models of sepsis stimulated by injection of high doses of LPS, massive cytokine production may be lethal [6]. The neutrophils, monocytes, and macrophages of septic patients develop a refractory state to subsequent LPS exposure and become incapable of producing cytokines at levels comparable to those prior to sepsis [7]. This state of unresponsiveness is termed endotoxin tolerance and prevents overstimulation from continuous exposure to LPS. However, endotoxin tolerant cells are unable to respond to subsequent TLR challenges leads to up to three weeks of immune paralysis in humans. During this time, significant morbidity occurs in hospitalized patients due to super-infections; therefore, determining mechanisms that regulate endotoxin tolerance is critical to identify therapeutics that could reverse LPS-induced immune paralysis.

Despite significant understanding of mechanisms that regulate TLR responses in general, we have a relatively limited understanding of mechanisms that contribute to the development or maintenance of LPS tolerance. Several proteins, including IL-1 receptor-associated kinase M (IRAK-M) [8], Src homology 2 (SH2) domain-containing inositol-5-phosphatase 1 (SHIP1) [9], and suppressor of cytokine signaling 1 (SOCS1) [10], participate in LPS tolerance. IRAK-M is an inhibitor of the IRAK-1/IRAK-4 cascade and mice deficient for IRAK-M demonstrate an increased inflammatory response to bacterial infection [8]. Moreover, IRAK-M-deficient macrophages are significantly impaired in the development of LPS tolerance [8]. SHIP1 inhibits LPS-induced activation of MAPKs and cytokines [11] and regulates IRAK-M expression during endotoxin tolerance development [12]. Furthermore, SHIP1-deficient Bone Marrow Macrophages (BMM) fail to develop LPS tolerance [9], although others have reported that SHIP1 may also promote TLR4-mediated cytokine production [13]. Similarly, SOCS-1 is required for tolerance [14] and negatively regulates TLR4 MyD88-dependent signaling [15] causing SOCS-1-deficient mice to be highly susceptible to LPS [10]. In spite of these advances, additional molecular mechanisms that regulate LPS tolerance are yet to be defined.

Downstream of tyrosine kinases (DOK) comprise a growing family of adaptor molecules that function to limit tyrosine kinase-mediated signaling downstream of numerous immunoreceptors, including TLRs [16]. The role of DOK proteins in regulating TLR responses has only been partially defined. DOK1 and DOK2 function as negative regulators of LPS-induced ERK activation and TNFα production in macrophages, but they fail to regulate other TLR responses mediated by TLR2, TLR3 or TLR9 [17]. DOK3 is also expressed in myeloid cells but has a unique cytoplasmic domain that does not bind to Ras GTPase like DOK1 or DOK2 [18], [19]. Moreover, previous reports in B cells demonstrate that DOK3 negatively regulates JNK activation, but not ERK, in response to B-cell receptor (BCR) stimulation. The structural and functional differences between DOK3 and other DOK isoforms suggest that DOK3 has non-redundant roles in regulating immunoreceptor and possibly TLR signaling [20], [21], [22]. The aim of this study was to determine the role of DOK3 in regulating TLR responses and endotoxin tolerance in macrophages.

## Results

### DOK3 Expression is Reduced during Macrophage Stimulation with LPS

**Figure 1:**
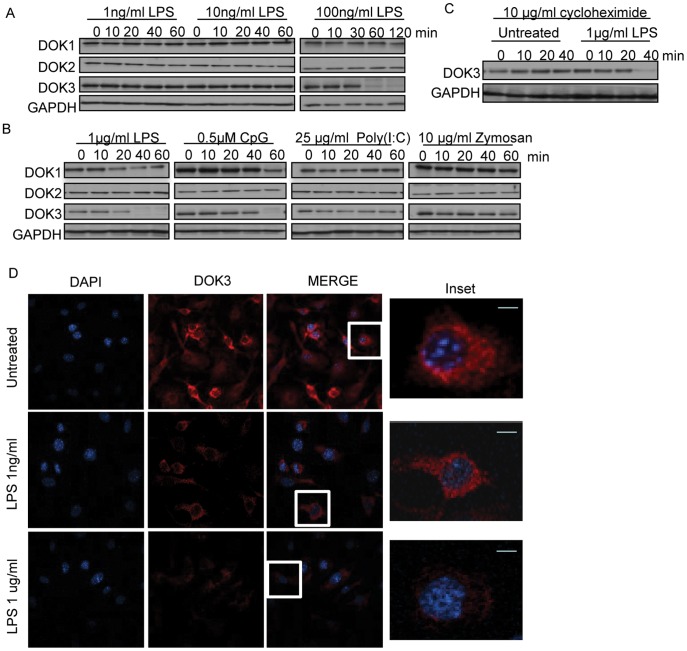
LPS stimulation of macrophages induces DOK3 degradation. BMM were stimulated with (A) 1, 10, or 100 ng/ml LPS; or (B) 1 µg/ml LPS (TLR4 ligand), 0.5 µM CpG (TLR9 ligand), 25 µg/ml Poly (I:C) (TLR3 ligand), or 10 µg/ml Zymosan (TLR2 ligand) for the indicated time and cell lysates were immunoblotted for DOK1, DOK2, DOK3, and GAPDH. (C) BMM were treated with 10 µg/ml of the translational inhibitor cycloheximide during LPS stimulation. Data are representative of three independent experiments. (D) BMM cells were stimulated with LPS (1 ng/ml and 1 µg/ml) for 1 h. The cells were fixed, permeabilized, and immunofluorescent staining for DOK3 (red) and DAPI (blue) was performed and visualized with a Leica TCS NT Confocal microscope. Bar is 10 µm.

DOK1 and DOK2 have been previously shown to participate in the negative regulation of LPS in macrophages [17]; however, the role of DOK3 has not been described. To assess the role of DOK3 in macrophage responses to TLR signaling, changes in DOK3 protein expression were examined following TLR stimulation of bone marrow macrophages (BMM). Whereas low doses of LPS (1 ng/ml and 10 ng/ml) failed to induce any changes in DOK proteins, higher doses of LPS (100 ng/ml or 1 µg/ml) significantly reduced the expression of DOK3 (Figure 1A and B). The LPS-triggered decrease in DOK3 expression was greater than decreases of other DOK isoforms. DOK1 expression was partially reduced at the highest dose of LPS in a manner similar to peritoneal macrophages [17]; whereas DOK2 expression remained stable at all doses (Figure 1B). We also tested additional TLR ligands, including ligands for TLR9 (CpG), TLR3 (poly (I:C)), and TLR2 (Zymosan) in the regulation of DOK3 expression. Although ligands for TLR3 or TLR2 failed to alter DOK3 expression, CpG triggered decreases in DOK3 expression with maximal loss at 60 min (Figure 1B) indicating that DOK3 is regulated downstream of TLR4 and TLR9 signaling in BMM.

Decreases in DOK3 expression induced by LPS occurred as early as 20 minutes but were maximal at 60 minutes suggesting that new protein synthesis might be required to regulate DOK3 expression. To investigate this possibility, we pretreated macrophages with the protein synthesis inhibitor, cycloheximide, prior to LPS stimulation. As seen in Figure 1C, DOK3 remained stable over 40 minutes in resting cells treated with cycloheximide but was significantly reduced after LPS stimulation even in the presence of cycloheximide. Thus, new protein synthesis was not necessary for decreases in DOK3 expression induced by LPS.

To determine whether LPS may induce DOK3 cellular translocation, localization of DOK3 after 1 hour of LPS stimulation was performed by confocal microscopy (Figure 1D). In resting cells, DOK3 was diffusely localized throughout the cytoplasm and the staining pattern remained stable following stimulation with a low dose of LPS (1 ng/ml). However, at doses of LPS that induce loss of DOK3 (1 µg/ml), we found DOK3 staining was significantly reduced and failed to be localized into discreet compartments. These results indicate that the observed reduction of DOK3 in cell extracts after LPS stimulation did not reflect sequestration of DOK3 into insoluble cellular compartments. Thus, degradation of DOK3 is the most likely explanation for DOK3 loss following treatment of cells with high dose of LPS.

### LPS-induces Degradation of DOK3 in a Phosphorylation Independent Manner

**Figure 2:**
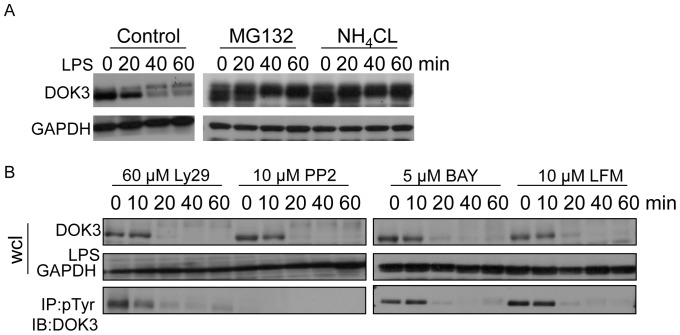
LPS stimulation in macrophages induces phosphorylation-independent degradation of DOK3. (A) BMM were stimulated with 1 µg/ml LPS and DMSO (control), proteasomal inhibitor (10 µM MG132), or lysosomal inhibitor (20 mM NH_4_Cl) or (B) BMM were stimulated with 1 µg/ml LPS in the presence of Src inhibitor (PP2), Syk inhibitor (Bay), PI3 kinase inhibitor (Ly294002), or Btk inhibitor (LFM) for the indicated time and cell lysates were immunoblotted for DOK3 and GAPDH. DOK3 phosphorylation was determined by immunoprecipitation (IP) with an anti-phosphotyrosine antibody and immunoblotting (IB) for DOK3. Data are representative of three independent experiments.

To determine whether LPS induces DOK3 degradation within proteosomes and lysosomes, BMM were treated with the proteosome inhibitor, MG132, or the lysosome inhibitor, NH_4_Cl. We found that both MG132 and NH_4_Cl blocked LPS-induced DOK3 degradation leading to the accumulation of a higher molecular weight DOK3 species (Figure 2A). These results imply that LPS induces the degradation of DOK3 within proteosomes and lysosomes, leading to reduced DOK3 expression.

The contribution of DOK3 phosphorylation to degradation was investigated by treating BMM with Ly294002 (PI3K inhibitor), PP2 (Src inhibitor), BAY 61–3606 (Syk inhibitor), or LFM-A13 (Btk inhibitor) for 30 minutes prior to LPS stimulation. Phosphorylated DOK3 was present in resting cells treated with inhibitors of PI3K, Syk and Btk but was not evident when Src family kinases were inhibited with PP2 (Figure 2B, bottom panel). These data suggest that DOK3 is constitutively tyrosine-phosphorylated by a member of the Src family of protein tyrosine kinases in resting macrophages. Furthermore, LPS-induced DOK3 degradation occurred in PP2 treated cells indicating that phosphorylation is not necessary for degradation (Figure 2B). Taken together, Src kinases participate in the phosphorylation of DOK3, but LPS-mediated DOK3 degradation is phosphorylation independent.

### LPS Induces Ubiquitination of DOK3

**Figure 3:**
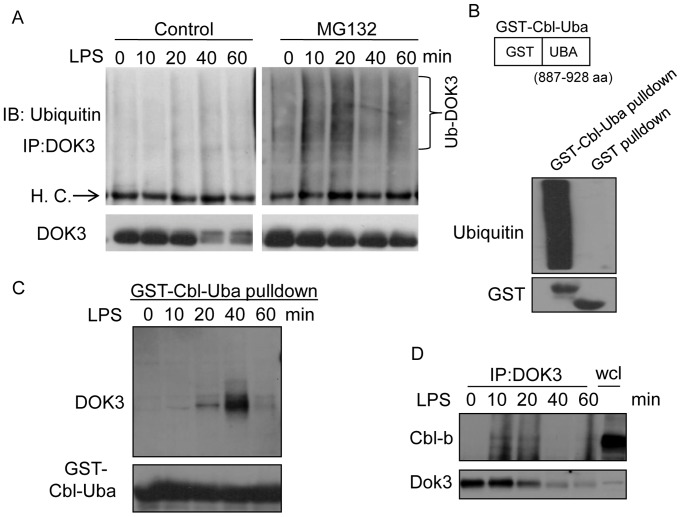
DOK3 is ubiquitinated through interaction with the E3 ligase Cbl-b in response to LPS stimulation. (A) BMM were stimulated with 1 µg/ml LPS ±10 µM MG132 for the indicated time, and ubiquitinated DOK3 was determined by immunoprecipitating with an anti-DOK3 antibody and immunoblotting with an anti-ubiquitin antibody. DOK3 immunoblot confirms degradation of DOK3 in control but not MG32 treated cells. H.C., heavy chain of IgG (B) GST-Cbl-Uba, comprised of GST and 887–928 amino acids (aa) of Cbl-b, was used to immunoprecipitate ubiquitinated proteins (bottom panel shows input amount of GST or GST-fusion). (C) GST-Cbl-Uba pulldown of LPS stimulated BMM lysates were immunoblotted for DOK3. Input amount of GST-fusion protein is the bottom blot. (D) DOK3 immunoprecipitation of LPS treated BMM immunoblotted for Cbl-b and DOK3. wcl, whole cell lysate Data are representative of three independent experiments.

To elucidate the molecular mechanism underlying LPS-induced degradation of DOK3, we examined the ubiquitination of DOK3 in response to LPS stimulation in the presence of MG132, which inhibits the degradation of ubiquitin-conjugated proteins. DOK3 was immunoprecipitated from LPS-stimulated BMM cell lysates and probed with antibodies to ubiquitin. The results show that in the presence of MG132, ubiquitinated DOK3 was observed as early as 10 minutes and peaked at 20 minutes after LPS stimulation (Figure 3A). Immunoblot of DOK3 confirmed that MG132 blocked the LPS-induced degradation seen in the control cells.

To verify that DOK3 was ubiquitinated, an ubiquitin-binding GST-fusion protein was used to co-precipitate DOK3. The ubiquitin binding domain of Cbl (Cbl-Uba) has a high binding affinity for ubiquitinated proteins [23], and a GST-Cbl-Uba (amino acids 887–928) fusion protein was generated to precipitate ubiquitinated proteins. Unlike GST alone, GST-Cbl-Uba fusion protein was able to pull down ubiquitinated proteins (Figure 3B) from LPS treated BMM and specifically precipitated DOK3 (Figure 3C). Co-precipitation was observed as early as 10 minutes and peaked at 40 minutes following LPS simulation (Figure 3C). These data suggest that LPS induces ubiquitination of DOK3, or the association of DOK3 with ubiquitinated proteins, and subsequent degradation of DOK3 in BMM.

Previous studies have indicated that LPS stimulation induces the activation of the E3 ligase Cbl-b, which is required to limit TLR signals [24], [25]. Therefore, taking a candidate approach, Cbl-b was investigated for association with DOK3 following LPS stimulation. Cbl-b co-immunoprecipitated with DOK3 at 10 to 20 min in LPS-stimulated BMM (Figure 3D) indicating that LPS stimulation promotes the association of DOK3 with the E3 ligase, Cbl-b. Taken together, these data suggest that LPS induces the association of Cbl-b and DOK3, with subsequent DOK3 ubiquitination and degradation.

### DOK3 Inhibits LPS-induced ERK Activation

**Figure 4:**
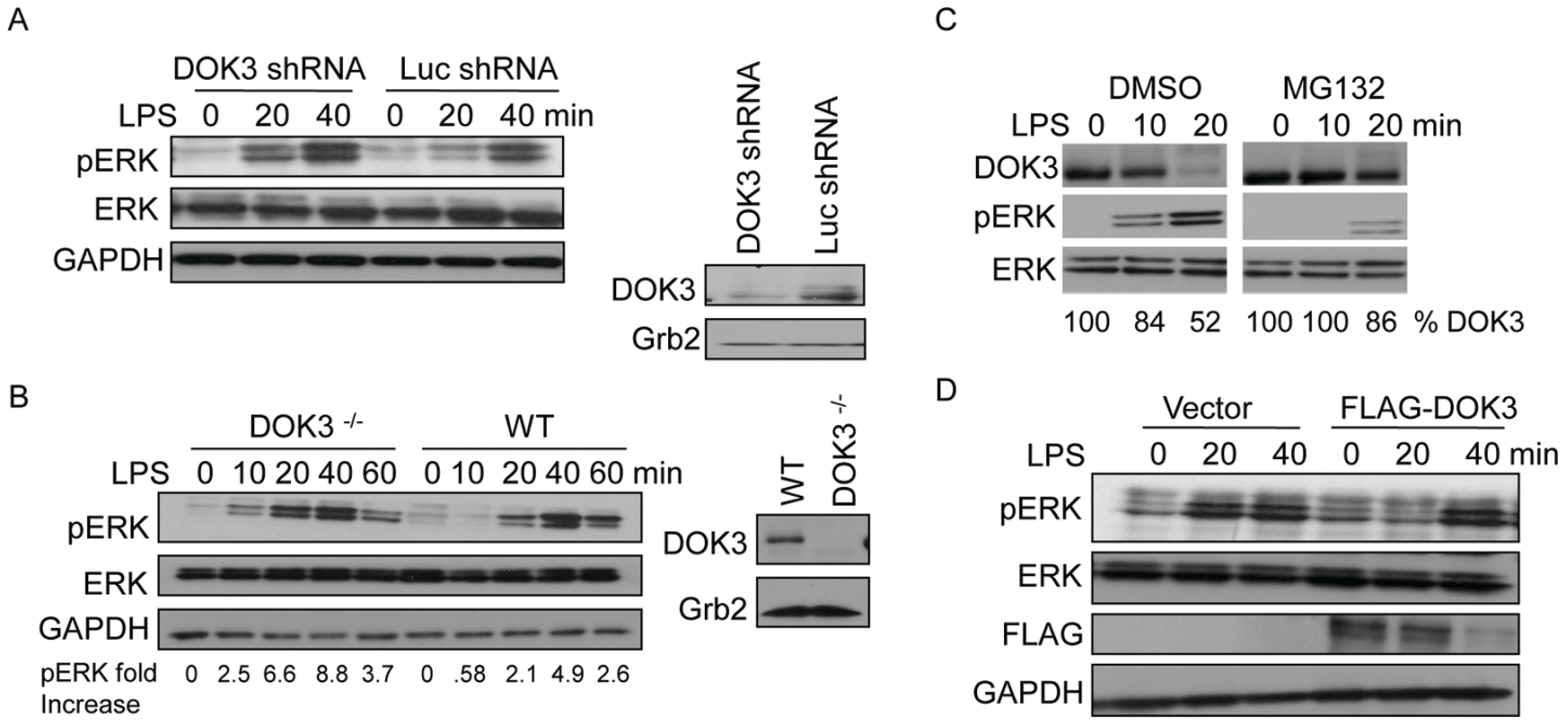
LPS-induced degradation of DOK3 mediates ERK activation. (A) RAW264.7 macrophages, transduced with 23 nt plus hairpin shRNA designed to knock down DOK3 or a control luciferase knock down, were lysed and decreased DOK3 expression was confirmed by immunoblotting. Cells were and stimulated with 1 µg/ml LPS for the indicated time and cell lysates were immunoblotted for pERK, ERK, and GAPDH. (B) Wild type (WT) or DOK3-deficient BMM were stimulated with 1 µg/ml LPS and cell lysates were immunblotted for pERK, ERK, and the GAPDH. The amount of DOK3 and Grb2 was detected by immunoblot in cell lysates. (C) BMM cells were pretreated with DMSO or 10 µM MG132 and stimulated with 1 µg/ml LPS for the indicated times. Lysates were immunoblotted for phosphorylated ERK (pERK), ERK, and DOK3. (D) RAW264.7 macrophages transfected with FLAG-tagged mouse DOK3- were stimulated with 1 µg/ml LPS for the indicated time and cell lysates were immunoblotted for pERK, ERK, FLAG and GAPDH. Data are representative of three independent experiments.

In HEK 293 cells, DOK3 has been shown to inhibit the Ras-Erk pathway by sequestering Grb2 downstream of protein-tyrosine kinases [19]. However, this function of DOK3 in regulating TLR signaling in macrophages has not been described. To determine the role of DOK3 in regulating ERK activation downstream of LPS, we generated a stable DOK3 knock-down in RAW264.7 macrophage cells with shRNA lentiviral transduction. Decreased DOK3 expression was confirmed by western blot of cell lysates of DOK3 knock down cells as compared to control luciferase shRNA (Figure 4A). In DOK3 knock-down cells, LPS stimulation induced earlier ERK activation compared to control cells (Figure 4A). To ensure that the increases in LPS-induced ERK activation were not due to off-target effects of the lentivirus, BMM were derived from DOK3-deficient mice [26] and stimulated with LPS. DOK3-deficient BMM demonstrated increased LPS-induced ERK activation compared to wild type macrophages (Figure 4B). These results suggest that DOK3 protein levels inhibit LPS-induced ERK activation.

To test this hypothesis, DOK3 degradation was blocked with MG132 prior to LPS stimulation, and ERK phosphorylation was determined by immunoblotting. In control cells treated with DMSO, LPS stimulation induced ERK activation in parallel to DOK3 degradation, with a peak at 20 minutes consistent with degradation of 50% of DOK3. Prevention of protein degradation by MG132, including DOK3, resulted in an inhibition of ERK activation (Figure 4C). Moreover, over-expression of DOK3 (FLAG-tagged DOK3) in RAW264.7 macrophage cells attenuated LPS-induced ERK activation (Figure 4D). Interestingly, LPS stimulation induced the degradation of over-expressed FLAG-DOK3, similar to degradation of endogenous DOK3, and ERK activation was delayed until 40 minutes, when FLAG-DOK3 was nearly degraded. Taken together, the expression of DOK3, as controlled by LPS-induced ubiquitination and degradation, regulates ERK activation.

### LPS Induces SOS1 Degradation in a DOK3-dependent Manner

**Figure 5:**
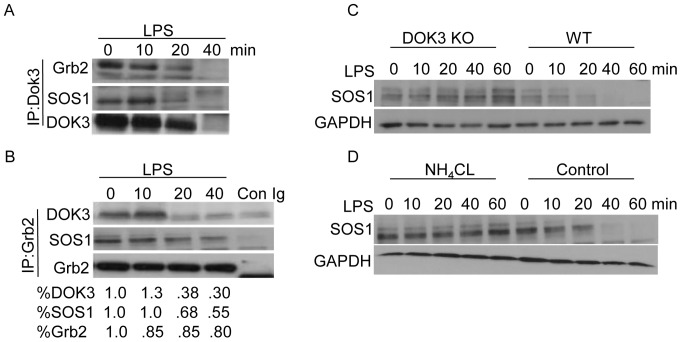
DOK3 associates with Grb2 and SOS1 and promotes SOS1 degradation in response to LPS. BMM were stimulated with 1 µg/ml LPS for indicated time and cell lysates were immunoprecipitated with (A) anti-DOK3 antibody or (B) anti-Grb2 antibody followed by immunoblotting for DOK3, Grb2, and SOS1. As a control, isotype matched antibody was used for immunoprecipitation (Con Ig). Protein levels were quantified using Image J. (C) Wild type and DOK3^−/−^ BMM were stimulated with 1 µg/ml LPS for indicated time and immunoblotted for SOS1 and GAPDH. (D) BMM were treated with NH_4_CL or PBS (control) and were stimulated with 1 µg/ml LPS and immunoblotted for SOS1 and GAPDH. Data are representative of three independent experiments.

In HEK293 cells, DOK3 serves to regulate Ras-ERK activation by binding to and sequestering Grb2 away from SOS1, a Ras guanine nucleotide exchange factor [19]. To address whether a similar mechanism was operative in LPS-stimulated BMM, DOK3 was immunoprecipitated and protein complexes immunoblotted for Grb2 and SOS1 (Figure 5A). In resting BMM and at 10 minutes after LPS stimulation, DOK3, Grb2, and SOS1 co-immunoprecipitated with the use of either anti-DOK3 (Figure 5A) or anti-Grb2 (Figure 5B) antibodies. At 20 minutes, significantly less DOK3 co-precipitated with Grb2 consistent with DOK3 degradation, while the association of GRB2 and SOS1 remained intact (Figure 5B). Interestingly, SOS1 also showed significant reductions after LPS stimulation that paralleled DOK3 degradation (Figure 5B). These findings suggest that LPS stimulation leads to reductions in SOS1 in addition to DOK3.

To address this hypothesis, SOS1 levels were evaluated in DOK3-deficient BMM after LPS stimulation. In wild type BMM, SOS1 levels were significantly reduced by 20 minutes and undetectable at 60 minutes after LPS treatment (Figure 5C). However, SOS1 levels remained stable after LPS treatment in DOK3-deficient BMM (Figure 5C). Therefore, SOS1 appears to be degraded in a DOK3-dependent manner after LPS stimulation. To evaluate this possibility, we treated wild type BMM with NH_4_CL to prevent lysosome-mediated protein degradation. NH_4_Cl treatment prevented SOS1 degradation in wild type cells similar to the stable SOS1 levels observed in DOK3-deficient cells (Figure 5D). Thus, LPS induces SOS1 degradation in the lysosome in a DOK3-dependent manner in BMM, a mechanism of SOS1 regulation not previously appreciated.

### DOK3 Alters LPS-induced Cytokine Production and Contributes to Survival in vivo

**Figure 6:**
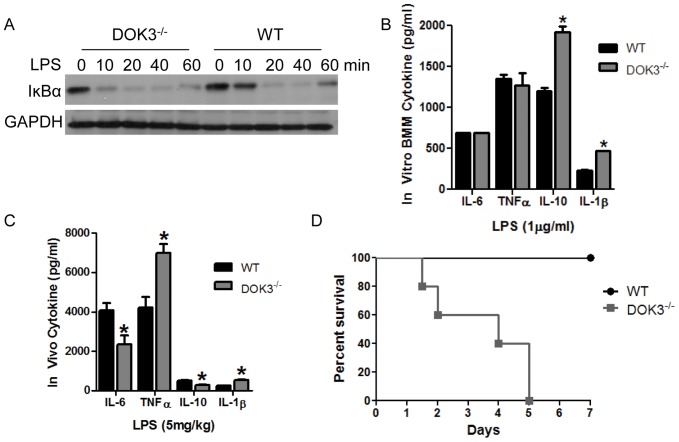
DOK3 alters LPS-induced inflammatory cytokine expression and is required for survival after LPS challenge. (A) Wild type and DOK3-deficient BMM were stimulated with 1 µg/ml LPS and cell lysates were immunoblotted for IκBα and GAPDH. (B) Wild type and DOK3-deficient BMM were treated with 1 µg/ml LPS for 16 h and amounts of IL-10, IL-6, TNFα, and IL-1β in conditioned media were determined by ELISA. (C) Serum was collected from WT and DOK3*^−/−^* mice prior to and 60 min. following challenge with 5 mg/kg LPS and serum IL-6, TNFα, IL-10 and IL-1β levels were determined by ELISA. (D) DOK3*^−/−^* mice are hypersensitive (P<0.0027) compared to WT mice following i.p. challenge with a sublethal LPS dose (70 mg/kg). The * in C and D indicates a significant difference (P<0.05) between cell type/strain and results are expressed as means ± S.D.

NF- κB activation is a critical component of TLR signaling and leads to the production of several cytokines. In order to measure the activation of NF-κB, the amount of IκBα was determined following LPS stimulation in wild type and DOK3-deficient BMM. In response to LPS, DOK3-deficient BMM showed more rapid and sustained degradation of IκBα compared to wild type cells (Figure 6A), indicating that DOK3 negatively regulates NF-κB activation downstream of LPS stimulation. TNFα, IL-6, IL-10 and IL-1β are important mediators of LPS-induced inflammation; therefore, these cytokines were measured in conditioned media from LPS-stimulated (1 µg/ml) wild type and DOK3-deficient BMM. While there was little difference in LPS-induced TNFα and IL-6 between strains, IL-10 and IL-1β production was significantly increased in DOK3-deficient BMM (Figure 6B). In sum, DOK3 negatively regulates NF-κB activation and the production of the pro-inflammatory cytokine IL-1β and the anti-inflammatory IL-10 following stimulation with LPS.

To investigate the role of DOK3 in vivo, wild type and DOK3-deficient mice were challenged with an intraperitoneal (i.p.) injection of 5 mg/kg LPS and blood was drawn prior to and 1 h following the administration of LPS. Interestingly, DOK3^−/−^ mice had a significantly increased TNFα and IL-1β response, while IL-6 and IL-10 levels were decreased in vivo compared to wt (Figure 6C). Furthermore, mice were challenged i.p. with a sublethal dose (70 mg/kg) of *Salmonella typhosa* LPS and monitored for survival over 7 days. All mice became ill but 100% of wild type mice recovered, while all DOK3-deficient mice succumbed (Figure 6D). Thus, DOK3 plays a role both in vitro and in vivo limiting LPS-induced cytokine responses and sepsis.

### DOK3 and SOS1 are Stably Expressed during Endotoxin Tolerance

**Figure 7:**
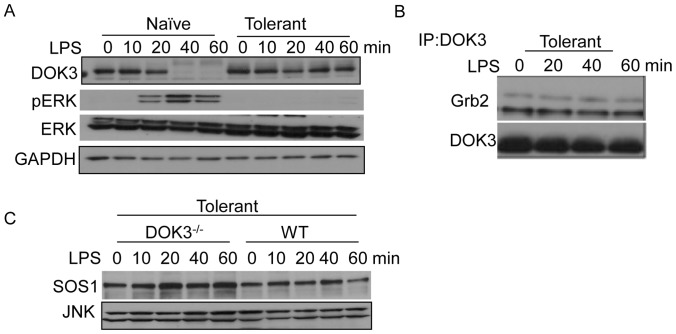
DOK3 and SOS1 are stable during LPS-induced tolerance. (A) BMM were pretreated with 1 µg/ml LPS for 18 hours, rested in fresh media for 2 hours and re-stimulated along with naïve cells with 1 µg/ml LPS for indicated time and cell lysates were immunoblotted for DOK3, pERK, ERK, and GAPDH. (B) Tolerant BMM were stimulated with 1 µg/ml LPS and cell lysates were immunoprecipitated with anti-DOK3 antibody and immunoblotted for Grb2 and DOK3. (C) Tolerant wild type and DOK3-deficient BMM were re-stimulated with 1 µg/ml LPS and lysates immunoblotted for SOS1 and JNK (loading control). Data are representative of three independent experiments.

An initial exposure to LPS induces a transient state of hypo-responsiveness to a subsequent challenge with LPS, termed endotoxin tolerance. To induce tolerance, BMM were pretreated with 1 µg/ml LPS, rested in fresh media, re-challenged with LPS, and ERK activation was evaluated to confirm tolerance. Compared to naïve cells where LPS induced ERK phosphorylation, re-stimulation of LPS-pretreated cells failed to activate ERK, indicating that the cells are tolerant towards LPS (Figure 7A). Unlike naïve cells stimulated with LPS, tolerant BMM re-stimulated with LPS maintain stable DOK3 expression.

To determine whether DOK3 remained associated with Grb2 in LPS-tolerant BMM, wild type tolerant BMM were re-challenged with LPS and lysates were immunoprecipitated with anti-DOK3 antibody. As compared to naïve cells (Figure 5B), tolerant wild type BMM re-challenged with LPS showed constitutive and stable association of Grb2 with DOK3 (Figure 7B). Additionally, SOS1 levels remained stable during tolerance (Figure 7C, WT). As compared to wild type tolerant BMM, SOS1 protein levels were further increased in DOK3-deficient LPS-tolerant BMM (Figure 7C). Taken together, DOK3 and SOS1 are stabilized during tolerance.

### DOK3–deficient BMM have Increased Sensitivity to Endotoxin Tolerance

**Figure 8:**
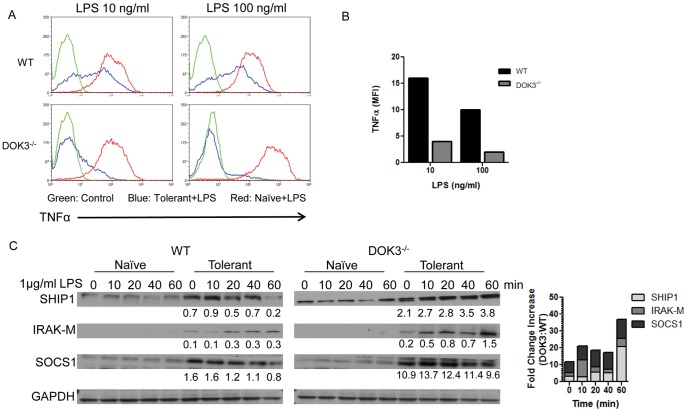
DOK3-deficient macrophages are more sensitive to endotoxin tolerance. Tolerance was induced in wild type and DOK3-deficient BMM with the indicated dose of LPS (10 or 100 ng/ml) overnight, followed by resting in fresh media for 2 h and re-challenged with the same dose of LPS for 12 h in the presence of Brefeldin A. Intracellular TNFα production was assessed with flow cytometry after the cells were fixed, permeabilized, and stained with anti-TNFα. Results are represented (A) by one-parameter histograms (green is control staining, red is naïve cells, and blue is tolerant cells) or (B) by MFI (mean fluorescence intensity) of TNFα-producing population and representative of two independent experiments. (C) Tolerant wild type and DOK3-deficient BMM or naïve cells were stimulated with 1 µg/ml LPS and immunoblotted for SHIP1, IRAK-M, SOCS1, and GAPDH. Densitometry of the bands was determined using image J normalizing to GAPDH, and the relative numerical value was written below the band. Data are representative of three independent experiments.

Because DOK3 levels remain stable during endotoxin tolerance, we next investigated the role of DOK3 in tolerance. WT and DOK3^−/−^ BMM were initially treated with 10 ng/ml or 100 ng/ml of LPS to induce tolerance. Cells were subsequently re-challenged with the same dose of LPS and intracellular TNFα levels were quantified by intracellular flow cytometry. WT BMM challenged with 10 ng/ml or 100 ng/ml of LPS displayed a partial suppression of TNFα after re-stimulation (Figure 8A, Blue) compared to naïve cells (Red). In contrast, DOK3-deficient BMM acquired LPS tolerance at low doses of LPS with suppression of TNFα expression at 10 and 100 ng/ml of LPS (Figure 8A and 8B). Thus, in the absence of DOK3, BMM are more sensitive to tolerance induction by LPS.

To investigate the molecular mechanism of enhanced tolerance in DOK3-deficient BMM, we assessed the expression of several mediators of endotoxin tolerance, including SH2-containing Inositol phosphatase-1 (SHIP1) [11], [12], Interleukin-1 receptor-associated kinase in myeloid cells (IRAK-M) [8], and Suppressor of cytokine signaling -1 (SOCS1) [10]. In WT and DOK3-deficient BMM, tolerance increased expression of SHIP1, IRAK-M, and SOCS1 compared to naïve cells, supporting their proposed role in tolerance (Figure 8C). Furthermore, comparing the quantified increased protein levels in tolerant cells compared to naïve levels indicates that DOK3-deficient tolerant BMM had substantially higher expression of SHIP1, IRAK-M, and SOCS1 expression compared to WT cells (Figure 8C). These findings suggest that in the absence of DOK3, BMM are more sensitive to LPS-induced ERK activation with subsequent upregulation of a gene expression profile that promotes endotoxin tolerance.

## Discussion

Innate immunity is critical for the first line of defense against bacterial and viral pathogens and is required to control early infections. TLR signaling stimulates the innate immune response by producing proinflammatory cytokines including TNFα, IL-6, and IL-1β, which leads to additional immune cell activation [27]. If left unchecked, the persistent cytokine responses to sustained TLR stimulation can lead to a cytokine storm resulting in sepsis and death. To prevent this result, persistent LPS stimulation can lead to a state of hypo-responsiveness to additional TLR stimuli. During this state, which can last up to 3 weeks in humans, the host is at high risk of super-infection with additional bacteria or virus. During sepsis, this state of immunosuppression is associated with substantial additional morbidity and mortality. Although some important regulators of tolerance have been indentified, our understanding of mechanisms by which tolerance is prevented or resolved are incompletely defined.

**Figure 9:**
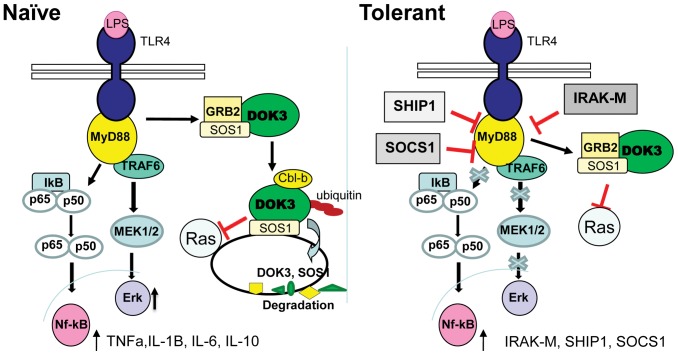
Proposed model of DOK3 regulation of TLR4 signaling and endotoxin tolerance. In naïve cells, DOK3 associates with Grb2 and SOS1 constitutively. Upon LPS stimulation, DOK3 becomes ubiquitinated, possibly by Cbl-b, and degraded, thereby releasing Grb2. SOS1 is also degraded in a DOK3-dependent manner thus limiting ERK activation. In LPS-induced tolerant cells, DOK3 and SOS1 expression remains stable during repeated LPS challenge. DOK3 may limit ERK activation during tolerance by binding to and sequestering Grb2 and SOS1. Mediators of tolerance including IRAK-M, SHIP1, and SOCS1 are upregulated.

We show here that DOK3 in macrophages has a non-redundant role in TLR signaling through TLR4 and TLR9 and that DOK3 has a regulatory role in preventing LPS induced tolerance. Our findings suggest a model in which LPS stimulation down-regulates the expression of DOK3 by targeting it for degradation via the ubiquitin-mediated pathway, and this process is independent of DOK3 phosphorylation (Figure 9). Moreover, SOS1 is targeted to the lysosome in a DOK3-dependent manner following LPS stimulation thus limiting ERK activation. Consequently, LPS induces enhanced ERK activation in the absence of DOK3. DOK3 also negatively regulates LPS induced NF-κB activation and the cytokines IL-1β and IL-10 in vitro and TNF and IL-1β in vivo. However, in vivo DOK3-deficient animals had decreased IL-6 and IL-10 production in response to LPS compared to wild type. While the contribution of LPS responses from in vitro data is representative of bone marrow derived macrophages, multiple immune cells including monocytes, peritoneal macrophages, bone marrow macrophages and B cells contribute in vivo. The fact that DOK1 and DOK2 are degraded by peritoneal macrophages in response to LPS [17] but differs here in bone marrow macrophages where we see a partial reduction in DOK1 but no change in DOK2 protein expression suggests that DOK3 has a non-redundant role in limiting TLR responses in a cell-type specific manner. Additionally, as compared to DOK1 and DOK2 that limit ERK activation but do not regulate NF-κB activation downstream of TLR4, DOK3 negatively regulates both ERK and NF-κB activation. Taken together, DOK3 represents a novel negative regulator of LPS signaling in macrophages.

The signal for ubiquitination of DOK3 is likely unique to TLR signaling. Generally, PTK-elicited phosphorylation events trigger protein degradation through the ubiquitination pathways [28]. For example, V-Src targets DOK1 for ubiquitin-mediated proteasomal degradation during cellular transformation [29]. Here we have shown that Src kinases phosphorylate DOK3 but that this phosphorylation event is not required for DOK3 degradation. Furthermore, DOK3 is constitutively phosphorylated in adherent macrophages possibly due to integrin activation [30], which is in contrast to B cells, where DOK3 phosphorylation occurs in response to BCR stimulation [20]. However, DOK3-phosphorylation may regulate protein-protein interactions in response to TLR signals. Ubiquitination and subsequent degradation of DOK3 has not been described in B cells; thus, ubiquitin-mediated degradation of DOK3 may be uniquely associated with TLR signaling or to the myeloid lineage.

DOK3 contributes to the regulation of ERK activation downstream of LPS signaling and the formation of a Grb2-SOS1-DOK3 complex appears to limit ERK activation. Our findings reveal a different mechanism of ERK regulation from a previous report showing that DOK3 over-expression in non-hematopoietic cells inhibits ERK activation downstream of phosphotyrosine kinases (PTK) by promoting the sequestering of Grb2, thus limiting Grb2 and SOS1-mediated activation of Ras [19]. Our data show that SOS1 levels decrease in parallel to DOK3 after LPS stimulation and that SOS1 is degraded in the lysosome in a DOK3-dependent manner after LPS stimulation. Additionally, SOS1 levels are increased in BMM-derived from DOK3-deficient mice suggesting that DOK3 negatively regulates SOS1 levels. We propose that the ubiquitination and degradation of DOK3 promotes the degradation of SOS1 thereby limiting ERK activation (Figure 9). Mechanistically, DOK3 could bring SOS1 into proximity to an E3 ligase or could shuttle SOS1 into the proteosome itself and is the subject of future studies. Regulation of Ras activation by SOS1 degradation represents a novel mechanism of Ras and ERK regulation. SOS1 mutations have recently been reported to occur in Noonan’s syndrome and Hereditary Gingival Fibromastosis Type 1 disease, both of which are associated with abnormal Ras activation [31]. Additionally, exposure to environmental pollutants and toxins significantly induces SOS1 expression and contributes to cellular proliferation in a manner analogous to Ras activation mutations [32]. We hypothesize that the activity and protein levels of SOS1 are tightly controlled during cellular states of environmental stimuli including LPS. DOK3 remains stable during tolerance and could serve to sequester SOS1 and Grb2 thereby limiting activation by a different mechanism than during the initial LPS response. Indeed, during endotoxin tolerance, stable DOK3 expression correlates with inhibition of ERK activation during LPS re-challenge, whereas DOK3-deficiency leads to enhanced LPS tolerance due to increased sensitivity to the initial LPS stimulation. Thus, ERK activation is regulated in part by the degradation of DOK3, as compared to DOK1 and DOK2 which negatively regulate ERK activity by recruiting Ras-GAP [17]. Alternatively, degradation of DOK3 may indirectly intersect the Tpl2-mediated ERK activation signaling pathway, as LPS stimulation induced more rapid degradation of IκBα in DOK3-deficient BMM compared to wild type, which can cause more NF-κB1 p105 degradation and the release of Tpl2 from the ABIN-2-NF-κB1-Tpl2 complex [25], [33].

Although DOK3 is required to limit initial ERK activation following LPS stimulation, it is not required for the development of endotoxin tolerance. Indeed, cells deficient in DOK3 showed increased sensitivity to LPS induced tolerance in vitro. The LPS tolerance in DOK3-deficient cells was accompanied by significant upregulation of SHIP1, IRAK-M, and SOCS1 suggesting that the enhanced ERK and NF-κB activation, induced by the initial LPS stimulation, primes the cells for rapid induction of tolerance upon re-challenge. IRAK-M deficiency leads to enhanced ERK activation and cytokine responses in naïve cells but prevents the induction of tolerance [8]. Likewise, SHIP1 is required for endotoxin tolerance [9]. Like DOK3, SHIP1 is also required to limit CpG induced cytokine responses in macrophages and is also critical for CpG induced tolerance [34]. Similarly, SOCS-1 is required for tolerance and mice deficient for SOCS1 are more highly susceptible to LPS than wild type [10], [14]. Additionally, in tolerant DOK3-deficient cells, SOS1 is significantly upregulated compared to wild type cells suggesting that DOK3 also negatively regulates SOS1 during tolerance. Thus, DOK3 negatively regulates the expression of key endotoxin tolerance effecter proteins.

Here we have shown that DOK3 also negatively regulates cytokine responses to LPS. In the naïve state, DOK3-deficiency leads to increased IL-1β and TNFα compared to wild type. Previous studies have demonstrated that LPS tolerance is associated with increased expression of IL-10 in vitro and suggest that anti-inflammatory IL-10 may contribute to endotoxin tolerance [33]. LPS increases IL-10 production in monocytes from septic patients as well as monocyte cell lines [33], [35]. IL-1β is also reported to induce tolerance in vivo, as mice treated daily with doses of IL-1β were refractory to LPS stimulation [36] and U937 LPS-tolerant macrophages were associated with an increased secretion of IL-1β [37]. We speculate that the enhanced TNFα, IL-10 and IL-1β contribute to the enhanced sensitivity of DOK3^−/−^ BMM to tolerance.

In sum, we have identified DOK3 as a negative regulator of LPS signaling in macrophages. LPS treatment induces DOK3 ubiquitination and degradation and SOS1 degradation leading to ERK activation. In the absence of DOK3, early ERK activation occurs as well as increased SOS1 expression and NF-κB activation. This data indicates that DOK3 is a proximal negative regulator of TLR signaling (Figure 9). Additionally, DOK3 may serve to prevent LPS tolerance by negatively regulating TNFα, IL-10, IL-1β, SHIP-1, IRAK-M, and SOCS1. DOK3 therefore plays a significant role in the maintenance of immune cell activation in response to TLR stimuli.

## Materials and Methods

### Mice

129S1/SvImJ mice were obtained from commercial vendors and housed at the Oklahoma Medical Research Foundation (OMRF). DOK3-deficient 129S1/SvImJ mice were kindly provided by Pier Paolo Pandolfi (Departments of Medicine and Pathology, Harvard Medical School) [26]. Animals were maintained according to the guidelines of the Association for Assessment and Accreditation of Laboratory Animal Care at OMRF.

### Reagents

The following antibodies were used: DOK1, DOK2, DOK3, Cbl-b, SHIP1 and SOS1 (Santa Cruz Biotechnology); GST, ERK, PERK, Grb2, IRAK-M, SOCS1, IκBα, and cleaved caspase3 (Cell Signaling Technology); GAPDH (Abcam); ubiquitin (Covance); 4G10-argarose conjugate (phosphotyrosine) and Flag M2 (Upstate Biotechnology); fluorescently-conjugated secondary antibodies (Invitrogen), PE-conjugated anti-TNFα (EBioscience). *Escherichia coli* O55:B5 LPS (Sigma) was used for in vitro assays and *Salmonella typhosa* (Sigma) LPS was used for the in vivo survival analysis.

### Macrophage Cell Culture

RAW 264.7 cells were purchased from ATCC and maintained in complete medium. Bone marrow macrophage (BMM) cells were flushed from bones and cultured as previously described [38]. After 2 days, non-adherent BMM cells were collected and transferred at a density of 5 ×10^6^ and cultured for an additional 2 to 5 days. shRNA lentiviral particles (Santa Cruz Biotechnology) were used according to the manufacturer’s protocol. Briefly, RAW264.7 macrophage cells were cultured to 50% confluence, the media was removed and replaced with 1 ml of Polybrene (5 µg/ml)/media mixture per well (for 12-well plate). The cells were infected by adding the 6 µl DOK3 shRNA lentiviral particles (1×10^6^ infectious units of virus (IFU)) and stable clones expressing the DOK3 shRNA were selected with Puromycin (5 µg/ml). pCMV-FLAG- DOK3 transfection was performed by nucleofection following the Amaxa protocol for RAW264.7 macrophage cells.

### Construction of cDNA Plasmids

pCMV-FLAG-DOK3 was constructed by subcloning mouse DOK3 cDNA (IMAGE No. 40126241) into pCMV-FLAG (Sigma). GST-CBL-Uba construct was made by subcloning the Uba domain (887–928 aa) of Cbl-b (IMAGE No. 40129800) into the glutathione S-transferase (GST) fusion protein expression vector pGEX4T3 by PCR (Open Biosystems). The generated constructs were confirmed by DNA sequencing.

### TLR Stimulation and Endotoxin Tolerance

BMM were stimulated with TLR ligands for the indicated time. Conditioned media and cell lysates collected. TNFα, IL-6, IL-10 and IL-1β in duplicate supernatants were measured by enzyme-linked immunosorbent assay (eBioscience). For tolerance, BMM were pretreated with 1 µg/ml LPS from *E. coli* for 18 hours, rested in fresh media for two hours, then re-stimulated with LPS. For intracellular TNFα staining, cells were stimulated for 12 h in the presence of 10 µg/ml Brefeldin A (Sigma), fixed and permeabilized, followed by blocking with 2.4G2 and staining with phycoerythrin-labeled antibody to TNFα (eBioscience). Cells were analyzed by flow cytometry (FACS Calibur).

### Immunoprecipitation

Cells lysates were prepared using ice-cold radioimmunoprecipitation assay (RIPA) buffer as previously described [39]. Immunoprecipitation were performed by incubating the precleared cell lysates with primary antibody on ice for 1 h and then adding protein A or G beads at 4°C for 1 h with rotation. The beads were washed and the immunoprecipitation complexes were dissolved in SDS-PAGE sample buffer for SDS-PAGE. The proteins were transferred to nitrocellulose or PVDF membranes, immunoblotted with the appropriate antibodies, and visualized with the enhanced chemiluminescence (ECL) system (Pierce).

### Expression, Purification and Pulldown Assays of GST Fusion Protein

GST alone or GST-Cbl-Uba fusion protein were expressed in *E. coli* (JM109) and purified by affinity glutathione-agarose beads as described previously [1]. The protein-bead complexes (15–20 µg protein) were incubated with cell lysates (∼1 mg) at 4°C for 2 hours with rotation. The beads were washed three times with RIPA buffer and dissolved in SDS-PAGE sample buffer for western blotting.

### Immunofluorescent Confocal Microscopy

BMM cells were in 8- or 16-well Permanox chamber slides (Costar) and serum starved for 3 h. Following stimulation for indicated time with LPS, cells were fixed with 1% paraformaldehyde in PBS, permeabilized with 0.05% saponin in PBS, blocking with antibody (2.4G2), and incubated with primary and secondary antibodies. Samples were viewed with either a Zeiss LSM510 (OMRF) or a Leica SP2 (OUHSC) confocal microscope and the images were analyzed with LSM confocal software.

### In vivo Assays

Wild type or DOK3^−/−^ mice (n = 5/group) were challenged i.p. with 70 mg/kg LPS in PBS and monitored twice daily for 7 days. Serum was collected by terminal heart puncture from wild type or DOK3^−/−^ mice (n = 3/group) one hour following i.p. challenge with 5 mg/kg LPS in PBS.

### Statistical Analysis

Statistical analyses were analyzed by Student’s t-test or survival analysis (Mantel-Cox Chi square) and performed with GraphPad Prism Plus software.
